# Burden, determinants and treatment status of metabolic syndrome among older adults in India: a nationally representative, community-based cross-sectional survey

**DOI:** 10.1136/bmjph-2023-000389

**Published:** 2023-11-20

**Authors:** Saurav Basu, Arun James Thirunavukarasu, Vansh Maheshwari, Mrunali Zode, Refaat Hassan

**Affiliations:** 1 Indian Institute of Public Health, Public Health Foundation of India, New Delhi, India; 2 School of Clinical Medicine, University of Cambridge, Cambridge, UK; 3 Oxford University Clinical Academic Graduate School, University of Oxford, Oxford, UK

**Keywords:** Public Health, Epidemiology, Community Health, Cardiovascular Diseases, Prevalence

## Abstract

**Introduction:**

Metabolic syndrome is a significance driver of mortality and morbidity in India, but nationally representative data regarding disease burden and treatment status are lacking. Here, a cross-sectional study was undertaken to establish national and regional estimates of disease burden and explore reasons for lack of treatment of component conditions of metabolic syndrome in Indian older adults (45 years and older).

**Methods:**

A cross-sectional study was undertaken using data from the first wave of the Longitudinal Ageing Study in India (2017–2018). Data for 66 606 individuals aged 45 years and above were analysed. The primary outcome was metabolic syndrome prevalence, defined by the National Cholesterol Education Programme ATP III criteria as an individual having any three of four component conditions: diabetes mellitus (DM), hypertension, abdominal obesity and hypercholesterolaemia. The secondary outcome of this study was treatment status of patients with component conditions.

**Results:**

Metabolic syndrome was found to have an overall weighted prevalence of 4.83% (n=3630, 95% CI 4.24 to 5.51). Females, urban residents, obese individuals and physically inactive people exhibited greater prevalence. The most prevalent component of metabolic syndrome was hypertension followed by abdominal obesity, DM and hypercholesterolaemia. 8.85% metabolic syndrome patient reported no treatment for component conditions, while 17.58% reported only partial treatment. Elderly individuals between 60 and 69 (crude relative risk ratios, cRRR 2.20, 95% CI 1.20 to 4.01) and 80 years and above (cRRR 7.48, 95% CI 1.99 to 28.16), urban residents (cRRR 2.45, 95% CI 1.48 to 4.05), those from richer monthly per capita consumption expenditure quintiles (cRRR 2.55, 95% CI 1.00 to 6.47) and those with additional comorbidities (cRRR 2.17, 95% CI 1.28 to 3.70) were more likely to report comprehensive treatment.

**Conclusions:**

This study highlights a substantial prevalence of metabolic syndrome in older adults in India and reveals remarkable disparities in provision of treatment. Better prevention, earlier detection and improved provision of treatment are urgently required to combat the rising prevalence of metabolic syndrome and reduce the burden of cardiovascular disease in India.

**WHAT IS ALREADY KNOWN ON THIS TOPIC**

- Metabolic syndrome is a significant source of cardiovascular mortality and morbidity.
- India has a high prevalence of the component conditions of metabolic syndrome, and consequently suffers disproportionately from the burden of downstream cardiovascular complications.
- Previous estimates of the prevalence of metabolic syndrome have generally been derived from single-centre clinical-based studies, with around 30% of adults in India estimated to be affected by metabolic syndrome.

**WHAT THIS STUDY ADDS**

- A large, nationally representative study of metabolic syndrome burden, treatment provision and risk factors for undertreatment in older adults.
- Overall weighted prevalence of metabolic syndrome in India estimated as 4.83%, likely an underestimate reflecting poor diagnosis rates. Almost one in four individuals with known metabolic syndrome in India are not treated for their component conditions.
- Adults are less likely to be treated for metabolic syndrome component conditions if aged 45–59 years, currently working, residing in a rural area, not exposed to news media, and having no other comorbidities.

**HOW THIS STUDY MIGHT AFFECT RESEARCH, PRACTICE OR POLICY**

- Improved access to diagnostic investigations is required to improve national prevalence estimates and allow patients with diabetes mellitus, hypertension and hypercholesterolaemia to be educated and seek treatment.
- Interventions aiming to address the disease burden of metabolic syndrome may specifically target risk factors for undertreatment identified in this study.

## Introduction

 Metabolic syndrome is a complex proinflammatory and prothrombotic state composed of a spectrum of disorders involving at least three of five interconnected metabolic disease states or abnormalities: hypertension (HTN), insulin resistance, diabetes mellitus (DM), central obesity and atherogenic dyslipidaemia.^1^ Multiple expert groups have produced definitions of metabolic syndromes including the WHO (1999) and the International Diabetes Federation (2005), with similar criteria varying with measurement modality and cut-offs.^2 3^ The National Cholesterol Education Programme (NCEP) ATP III definition of metabolic syndrome requires three or more of the following five criteria: waist circumference ≥40 inches in men or ≥35 inches in women, blood pressure ≥130/85 mm Hg, fasting triglyceride level ≥150 g/dL, fasting high-density lipoprotein (HDL) level <40 mg/dL in men or <50 mg/dL in women and fasting blood sugar level ≥100 mg/dL.^4^

The burden of cardiovascular mortality and morbidity is increasing worldwide, particularly in developing countries experiencing epidemiological, demographic, nutritional, social and economic transition.^5^ Increasing longevity, more sedentary lifestyles, high consumption of calorie dense foods and sugars with reduced intake of fresh fruits and vegetables, and rising obesity rates from childhood onwards are contributing to this noncommunicable disease pandemic, especially in South Asia.^6 7^ These increases in mortality and morbidity may be a consequence in part of an increasing rate of metabolic syndrome. However, parsing the independent effects of metabolic syndrome and other risk factors implicated in cardiovascular disease is complicated. For instance, metabolic syndrome is associated with older age, but this is also an independent risk factor of DM, HTN and other noncommunicable diseases.^8^ Furthermore, people with obesity in terms of both body mass index (BMI) and abdominal obesity are five times more likely to develop metabolic syndrome; but obesity comprises one of the diagnostic criteria for metabolic syndrome.^9^ Similar issues arise with other risk factors for metabolic syndrome, both non-modifiable (eg, ethnicity, sex) and modifiable (eg, physical activity, tobacco use and alcohol consumption).^10 11^ Nevertheless, metabolic syndrome is increasingly recognised as an independent risk factor of mortality and morbidity attributed to cardiovascular disease, myocardial infarction and stroke.^12^ Furthermore, people with metabolic syndrome are also at an increased risk of type 2 DM.^13^ It is estimated that people with metabolic syndrome experience a doubled risk of mortality from cardiovascular disease.^14^ Metabolic syndrome is a high-risk state because the complex multimorbid condition requires optimal levels of medication and lifestyle adherence to maintain desirable health outcomes and prevent the onset and progression of complications such as cardiovascular disease.^15 16^

India, with an estimated population of 1.4 billion people, has a very high burden of DM, HTN and abdominal obesity relative to rest of the world, thereby predisposing most of its older and elderly population to metabolic syndrome.^17 18^ Furthermore, poor medication adherence in a majority of patients on antidiabetes and/or antihypertensive medications and/or other cardiovascular medications have been reported in multiple systematic reviews that potentially may further worsen health outcomes.^19 20^ Lack of initiation of effective antidiabetes, antihypertensive and lipid lowering medications is a major component of poor treatment coverage in LMICs.^21^ For reference, the global burden of metabolic syndrome varies from 10% to 84% depending on the diagnostic criteria applied.^22^,^24^ In India, one systematic review reported a pooled estimate metabolic syndrome prevalence as 30% while an estimate from secondary data analysis among young and middle-aged adults reported a very low prevalence (1.1% in men and 1.5% in women).^25 26^ However, prior studies of metabolic syndrome prevalence in India have lacked national level representativeness, report from small sample sizes and are mostly facility-based studies with limited generalisability. In addition, little information is available regarding the treatment status of metabolic syndrome patients, and risk factors associated with lack of provision of treatment. Finally, the extent of undiagnosed metabolic syndrome in India is also unclear.

Accurate estimation of the burden, risk factors and treatment status of metabolic syndrome in high-risk groups such as older adults (aged 45 years and above) living in India is essential knowledge to develop effective public health policies and design interventions to improve health at the population level. Here, a nationally representative cross-sectional study was undertaken to determine the prevalence, predictors and treatment status of metabolic syndrome in older adults and elderly people in India.

## Methods

### Study design and sample

A cross-sectional analysis was undertaken using survey data obtained through the Longitudinal Ageing Study in India (LASI), first wave, which is a nationally representative survey of over 72 000 individuals aged 45 and above along with their spouses (regardless of age) across all states and union territories of India. LASI adopted a multistage stratified area probability cluster design for sampling and aimed to provide scientific evidence on the overall health status and socioeconomic well-being of India’s older population. The detailed methodology and information on the survey design and data collection is reported elsewhere.^27^ As some individuals (n=6790) under the age of 45 years were interviewed as part of LASI, this study was conducted using data from study participants aged ≥45 years only.

### Outcome and predictive variables

The primary outcome was metabolic syndrome prevalence. For the purposes of this study, metabolic syndrome was defined with NCEP ATP III criteria as an individual having any three of four component conditions: DM, HTN, abdominal obesity and hypercholesterolaemia.^28^ Abdominal obesity was estimated through measurement of waist circumference and defined as ≥35 inches for females and ≥40 inches for males, using a Gulick tape and adhering to an established protocol by trained field investigators.^27^ Self-reported status of DM, HTN and hypercholesterolaemia were assessed using the question, ‘Has any health professional ever diagnosed you with the following chronic conditions or diseases?’ with responses coded as ‘no’ or ‘yes’. Furthermore, to screen for undiagnosed cases of HTN, blood pressure was ascertained through measurement with an electronic blood pressure monitor by trained field investigators. New cases of HTN were considered as those individuals having a mean of last two readings of systolic blood pressure ≥140 mm Hg and/or diastolic blood pressure ≥90 mm Hg. Therefore, the individuals were categorised as hypertensive if they either self-reported the condition (previously diagnosed) or from high blood pressure detected during screening of the apparently healthy (newly diagnosed cases).

The secondary outcome of this study was treatment status of metabolic syndrome patients. The treatment status of DM, HTN and high cholesterol was assessed using the questions, ‘In order to treat or control your diabetes or high blood sugar, are you currently taking medications?’, ‘In order to control your blood pressure or HTN, are you currently taking any medication?’ and ‘Do you regularly take medications to help lower your cholesterol?’ respectively. Subsequently, the treatment status of individuals with metabolic syndrome was classified into three groups: no treatment (not on treatment for pre-existing DM or HTN or high cholesterol), partial treatment (on treatment for either DM or HTN or high cholesterol but not all conditions), and full treatment (taking treatment for the conditions present among DM, HTN or high cholesterol) (table 1). Further, the prevalence and risk factors of metabolic syndrome were assessed among individuals at higher risk, that is, those having either DM, HTN, high cholesterol or high BMI (≥25.0 kg/m^2^).^12^

**Table 1:** Classification of treatment seeking behaviour among participants having MetS conditions

MetS related conditions present	Treatment initiation	Treatment seeking behaviour
AO, DM, HC	Both DM and HCOnly 1 among DM and HCNone	Full treatmentPartial treatmentNo treatment
AO, DM, HTN	Both DM and HTNOnly 1 among DM and HTNNone	Full treatmentPartial treatmentNo treatment
AO, HTN, HC	Both HTN and HCOnly 1 among HTN and HCNone	Full treatmentPartial treatmentNo treatment
DM, HTN, HC	All 3 conditionsAny 1 or 2 of the conditionsNone	Full treatmentPartial treatmentNo treatment
AO, DM, HTN, HC	All 3 conditionsAny 1 or 2 of the conditionsNone	Full treatmentPartial treatmentNo treatment

AO, abdominal obesityDM, diabetes mellitus; HC, high cholesterol; HTN, hypertensionMetSmetabolic syndrome

Several potential biomedical and psychosocial risk factors were included in the predictive models (online supplemental table 1). These included sociodemographic variables such as sex, age, education, working status, place of residence and marital status. Monthly per capita consumption expenditure (MPCE) quintiles were calculated using standardised overall expenditure (food and non-food) and used as a summary measure of consumption. For modelling, MPCE was classified as poorest, poorer, middle, richer and richest quintiles. BMI was according to the WHO Pan Asian classification system.^29^ Media exposure was assessed based on the self-reported frequency of watching TV/listening to radio, reading books/newspapers/magazines and using a computer. Physical activity was assessed by the question, ‘How often do you take part in sports or vigorous activities, such as running or jogging, swimming, going to a health centre or gym, cycling, or digging with a spade or shovel, heavy lifting, chopping, farm work, fast bicycling, cycling with loads?’ Additional comorbidities were classified as binary variables, based on the presence or lack thereof of self-reported diseases such as cancer, chronic lung disease, chronic heart disease, stroke, arthritis and neurological problems. In addition, smoking status and alcohol consumption were recorded.

### Statistical analysis

Descriptive statistics and bivariate analysis were applied to describe sociodemographic information and lifestyle characteristics of individuals with metabolic syndrome. Individual weights were used throughout to make the estimates nationally representative. Multivariable binary logistic regression was carried out to estimate the OR of explanatory variables on the presence of metabolic syndrome, without controlling for sociodemographic factors as well as after controlling for those factors. Furthermore, multinomial logistic regression was performed to assess the treatment-seeking behaviour among participants having metabolic syndrome. Variables that had a significant association (p<0.05) with the outcome were added to the final adjusted model. Both crude and adjusted relative risk ratios (c/aRRR) were reported. LASI survey weighting was applied to compensate for the complex survey design. All regression models underwent diagnostic checks for multicollinearity and outliers. Estimates were presented with respective 95% CI and p values. Statistical significance was concluded where p values lay under 0.05 and where CI did not cross the null point. Data were analysed by using Stata V.15.1 (StataCorp).^27^

## Results

### Participants

**Figure 1:**
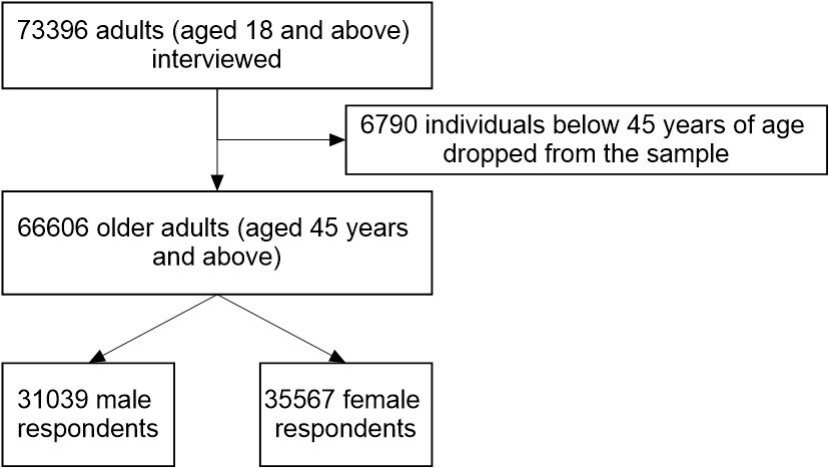
Flow chart depicting the inclusion process for this cross-sectional survey.

After ineligible patients were excluded, the total sample size was 66 606 (figure 1). Median age was 59 years; the male:female ratio was 0.87. The cohort was generally representative of the Indian population, in-keeping with the aims of LASI.

### Prevalence

The overall weighted prevalence of metabolic syndrome was 4.83% (n=3630, 95% CI 4.24% to 5.51%) and was higher in females (6.77%) relative to males (2.56%) (χ^2^=635.79, p<0.001). The weighted prevalence of self-reported DM and hypercholesterolaemia was 12.34% (n=8564, 95% CI 11.54% to 13.20%) and 2.28% (n=2310, 95% CI 2.07% to 2.51%), respectively. Weighted prevalence of HTN was found to be 48.79% (n=31 850, 95% CI 47.87% to 49.71%), including 27.53% previously diagnosed and 27.27% newly diagnosed hypertensives. Among the participants, 0.32% (n=432, 95% CI 0.27% to 0.37%) had all the four conditions of abdominal obesity, DM, HTN and hypercholesterolaemia, while 4.52% (n=3198, 95% CI 3.93% to 5.19%) had any three of these conditions.

The sociodemographic characteristics of the participants and distribution of metabolic syndrome are reported in table 2. Of the individuals with metabolic syndrome, a majority were females (75.66%), currently married (66.61%), not working (54.94%) and residing in urban areas (63.69%). Around four in five were obese (81.29%) and were not involved in any physical activity (74.88%). Fewer than half had one or more comorbidities (48.98%). The most prevalent metabolic syndrome component in individuals with the condition was HTN (98.55%, 95% CI 97.84% to 99.03%), followed by abdominal obesity (94.57%, 95% CI 93.22% to 95.66%), DM (89.40%, 95% CI 87.21% to 91.24%) and hypercholesterolaemia (25.47%, 95% CI 21.84% to 29.48%).

**Table 2:** Sociodemographic and lifestyle characteristics of participants with metabolic syndrome

Characteristics	Having MetS (N=3630)n (weighted %)	P value*
Age (years)		0.0067
45–59	1569 (39.97)	
60–69	1336 (39.18)	
70–79	595 (17.47)	
80 and above	130 (3.37)	
Sex		<0.001
Male	965 (24.34)	
Female	2665 (75.66)	
Education (n=2542)		0.0091
No education or less than primary	444 (15.84)	
Primary complete	611 (20.40)	
Secondary	971 (44.06)	
Higher	221 (6.72)	
Graduate and above	295 (12.98)	
Marital status		0.0412
Never married	29 (0.53)	
Currently married	2539 (66.61)	
Widowed/divorced/separated/deserted	1062 (32.86)	
Work status (n=1890)		<0.001
Not working	1101 (54.94)	
Currently working	789 (45.06)	
Place of residence		<0.001
Rural	1374 (36.31)	
Urban	2256 (63.69)	
MPCE quintile		<0.001
Poorest	438 (11.44)	
Poorer	519 (13.12)	
Middle	672 (15.69)	
Richer	870 (26.52)	
Richest	1131 (33.23)	
BMI (kg/m^2^) (n=3561)		<0.001
Underweight	19 (0.42)	
Normal	276 (5.93)	
Overweight	496 (12.36)	
Obese	2770 (81.29)	
Smoking status (n=281)		<0.001
No	155 (50.84)	
Yes	126 (49.16)	
Alcohol consumption (n=3620)		<0.001
No	3255 (92.54)	
Yes	365 (7.46)	
Physical activity		<0.001
Frequently	624 (19.37)	
Rarely	227 (5.75)	
Never	2768 (74.88)	
Media exposure		<0.001
No	475 (14.97)	
Yes	3128 (85.03)	
Additional comorbidities		<0.001
None	2068 (51.02)	
1 or more	1562 (48.98)	

* P- values denote the level of significance of Pearson’s chi-squareχ2 statistic.

BMI, body mass indexMetS, metabolic syndrome; MPCE, monthly per capita expenditure

**Figure 2:**
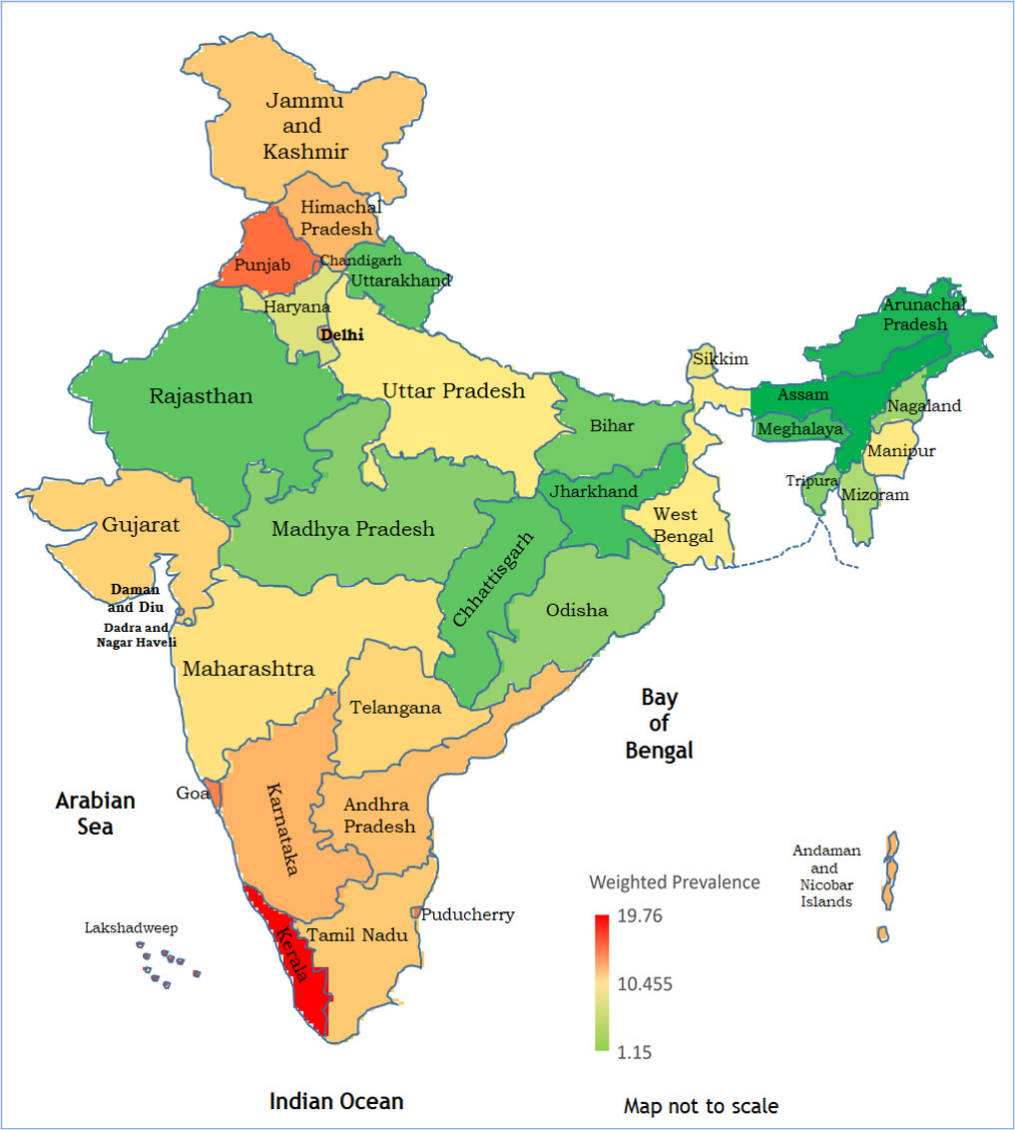
Heat map of the weighted prevalence of metabolic syndrome in Indian states. Weighted prevalence was calculated by applying appropriate state-level weights.

Considerable regional variation was observed in the prevalence of metabolic syndrome in India with the maximum prevalence reported in Kerala (19.76%), followed by Lakshadweep (15.86%), Punjab (12.44%) and Chandigarh (11.53%) while lowest prevalence was found in the North Eastern states of Assam (1.15%), Arunachal Pradesh (1.48%) and Meghalaya (1.87%) (figure 2, online supplemental table 2).

### Risk factors for metabolic syndrome

The factors associated with prevalence of metabolic syndrome among individuals at higher risk (those with one or more of DM, HTN, hypercholesterolaemia and high BMI≥25.0 kg/m^2^) (n=40 041) were assessed using two different models (table 3). On adjusting for all variables (model 1), it was observed that among those at higher risk, individuals aged 60–69 years (aOR 1.85, 95% CI 1.01 to 3.37), urban residents (aOR 3.34, 95% CI 2.02, 5.53), obese (aOR 11.4, 95% CI 5.91 to 22.04) and those with additional comorbidities (aOR 2.46, 95% CI 1.56 to 3.89) had significantly higher odds of having metabolic syndrome than their counterparts. On using stepwise backward elimination (model 2), age 60–69 years, females, urban residents, higher BMI and additional comorbidities were again the predictors significantly associated with higher likelihood of having metabolic syndrome in high-risk individuals.

**Table 3:** Predictors associated with prevalence of metabolic syndrome among individuals at higher risk*

Variables	Having MetS (N=3630)					
	Crude OR (95% CI)	P value	Model 1† aOR (95% CI)	P value	Model 2‡ aOR (95% CI)	P value
Age (years)		0.0008		0.0436		
45–59	Ref		Ref		Ref	0.0016
60–69	1.57 (1.19 to 2.07)§		1.85 (1.01, 3.37)§		1.90 (1.21 to 2.98)§	
70–79	1.30 (0.79 to 2.15)		2.21 (0.95, 5.12)		2.32 (1.30 to 4.12)§	
80 and above	0.69 (0.46 to 1.02)		0.51 (0.09, 2.91)		0.52 (0.16 to 1.73)	
Sex		<0.001		0.148		<0.001
Male	Ref		Ref		Ref	
Female	2.46 (1.93 to 3.13)		2.99 (0.68, 13.19)		2.68 (1.56 to 4.62)	
Education		0.0667				
No education or less than primary	Ref		Ref	0.1743	–	
Primary complete	1.09 (0.84 to 1.41)		1.35 (0.66, 2.78)			
Secondary	1.82 (1.18 to 2.79)§		0.68 (0.35, 1.31)			
Higher	0.95 (0.69 to 1.3)		0.58 (0.25, 1.33)			
Graduate and above	1.23 (0.79 to 1.93)		0.67 (0.30, 1.49)			
Marital status		0.1675		0.7137		
Never married	Ref		Ref		–	
Currently married	1.5 (0.76 to 2.98)		0.55 (0.06, 4.62)			
Widowed/divorced/separated/deserted	1.99 (0.92 to 4.29)		0.42 (0.04, 4.15)			
Work status		<0.001		0.912		
Not working	Ref		Ref		–	
Currently working	0.59 (0.47 to 0.74)		0.97 (0.54, 1.74)			
Place of residence		<0.001		<0.001		<0.001
Rural	Ref		Ref		Ref	
Urban	2.97 (2.35 to 3.74)		3.34 (2.02, 5.53)		2.71 (1.81 to 4.06)	
MPCE quintile		<0.001		0.2006		
Poorest	Ref		Ref		–	
Poorer	1.07 (0.84 to 1.36)		1.26 (0.55, 2.9)			
Middle	1.28 (0.97 to 1.67)		0.67 (0.29, 1.59)			
Richer	2.22 (1.51 to 3.27)**		0.74 (0.36, 1.53)			
Richest	2.75 (1.94 to 3.89)**		1.36 (0.68, 2.7)			
BMI (kg/m^2^)		<0.001		<0.001		<0.001
Underweight/normal	Ref		Ref		Ref	
Overweight	6.34 (4.91 to 8.20)¶		5.84 (2.54, 13.42)¶		5.43 (2.69 to 10.95)¶	
Obese	13.38 (10.36 to 17.29)¶		11.41 (5.91, 22.04)¶		10.92 (6.44 to 18.51)¶	
Smoking status		<0.001		0.005		<0.001
No	Ref		Ref		Ref	
Yes	0.35 (0.24 to 0.51)		0.51 (0.31, 0.82)		0.45 (0.30 to 0.67)	
Alcohol consumption		<0.001		0.334		
No	Ref		Ref		–	
Yes	0.48 (0.38 to 0.60)		1.25 (0.79, 1.99)			
Physical activity		<0.001		0.0750		0.1218
Frequently	Ref		Ref		Ref	
Rarely	1.06 (0.77 to 1.46)		2.62 (1.14, 6.04)§		2.03 (0.95 to 4.34)	
Never	1.77 (1.31 to 2.40)¶		1.47 (0.84, 2.57)		1.43 (0.90 to 2.26)	
Media exposure		<0.001		0.527		
No	Ref		Ref		–	
Yes	2.37 (1.87 to 3.02)		1.33 (0.55, 3.25)			
Additional comorbidities		<0.001		<0.001		<0.001
None	Ref		Ref		Ref	
1 or more	2.35 (1.78 to 3.11)		2.46 (1.56, 3.89)		2.37 (1.59 to 3.52)	

Goodness of fit: Mmodel 1, Pp=0.8433 and Mmodel 2, Pp=0.2679.

* Denominator (N=40 041) taken as individuals on higher- risk, that is, cases having DM or HTN or HC or high BMI (≥25.0).

† Model 1=all variables.

‡ Model 2=for significant variables using backward stepwise regression.

§ P<0.05, **P.

¶ P<0.001.

aOR, adjusted OR; BMI, body mass index; DMdiabetes mellitusHChigh cholesterolHTNhypertensionMetS, metabolic syndrome; MPCE, monthly per capita expenditure

### Treatment status of metabolic syndrome and their risk factors

The proportion of individuals having metabolic syndrome with a previous diagnosis of DM and/or HTN reporting a lack of initiation of treatment, on partial treatment and full treatment were 8.85% (95% CI 6.99% to 11.15%), 17.58% (95% CI 14.09% to 21.72%) and 73.56% (95% CI 68.52% to 78.06%), respectively. The factors associated with treatment status among participants with metabolic syndrome (n=3191) are reported in table 4. Increasing age was significantly associated with individuals being on partial treatment rather than taking no treatment. For instance, individuals aged 60–69 (cRRR 2.20, 95% CI 1.20 to 4.01) and 70 years and above (cRRR 2.13, 95% CI 1.14 to 4.00) were more likely to receive partial treatment than taking no treatment for their metabolic syndrome component conditions. Similarly, the likelihood of those aged >60 years receiving full treatment for their conditions was significantly higher relative to comparatively younger participants. Furthermore, urban residents (cRRR 2.45, 95% CI 1.48 to 4.05), those belonging to richer MPCE quintiles (cRRR 2.55, 95% CI 1.00 to 6.47), those having media exposure (cRRR 2.27, 95% CI 1.32 to 3.91) and individuals with additional comorbidities (cRRR 2.17, 95% CI 1.28 to 3.70) were more likely to report full treatment for their metabolic syndrome component conditions. In adjusted multinomial logistic regression analysis, those aged 60–69 years (aRRR 1.85, 95% CI 1.13 to 3.02), urban residents (aRRR 2.16, 95% CI 1.37 to 3.41), those reporting media exposure (aRRR 1.92, 95% CI 1.19 to 3.11) and individuals with additional comorbidities (aRRR 1.95, 95% CI 1.22 to 3.10) were more likely to be on full treatment for their component conditions of metabolic syndrome.

**Table 4:** Treatment-seeking behaviour among participants with metabolic syndrome (N=3191)

Variables	Not on Treatmentn (weighted %)(N=304)	Partial Treatmentn (weighted %)(N=662)	Full Treatmentn (weighted %)(N=2225)	Crude RRR^1^(95% CI)	Crude RRR^2^(95% CI)	Adjusted RRR^1^(95% CI)	Adjusted RRR^2^(95% CI)
Age (years)							
45–59	166 (54.56)	300 (35.56)	886 (37.25)	Ref	Ref	Ref	Ref
60–69	90 (29.81)	233 (42.68)	869 (40.56)	2.20 (1.20 to 4.01)*	1.99 (1.13 to 3.51)*	2.15 (1.16 to 3.98)*	1.85 (1.13 to 3.02)*
≥70	48 (15.64)	129 (21.76)	470 (22.20)	2.13 (1.14 to 4.00)*	2.08 (0.93 to 4.65)	2.05 (1.10 to 3.82)*	1.86 (0.98 to 3.51)
Sex							
Male	73 (20.82)	174 (30.75)	566 (22.23)	Ref	Ref	–	–
Female	231 (79.18)	488 (69.25)	1659 (77.77)	0.59 (0.30 to 1.18)	0.92 (0.55 to 1.54)		
Education (n=2222)							
No education or less than primary	33 (17.76)	97 (25.86)	264 (13.73)	Ref	Ref	–	–
Primary complete	58 (28.73)	104 (17.75)	379 (19.29)	0.42 (0.19 to 0.94)*	0.87 (0.42 to 1.81)		
Secondary	70 (32.49)	176 (29.14)	596 (49.25)	0.62 (0.29 to 1.31)	1.96 (0.84 to 4.56)		
Higher	17 (6.10)	45 (8.423)	131 (6.006)	0.95 (0.32 to 2.80)	1.27 (0.48 to 3.35)		
Graduate and above	19 (14.92)	48 (18.84)	185 (11.73)	0.87 (0.18 to 4.15)	1.02 (0.36 to 2.88)		
Marital status							
Never married	1 (0.0567)	8 (0.4616)	14 (0.3711)	Ref	Ref	–	–
Currently married	215 (69.65)	476 (72.93)	1537 (62.8)	0.13 (0.01 to 1.34)	0.14 (0.02 to 1.17)		
Widowed/divorced/separated/deserted	88 (30.29)	178 (26.6)	674 (36.83)	0.11 (0.01 to 1.13)	0.19 (0.02 to 1.68)		
Work status (n=1645)							
Not working	83 (54.4)	225 (52.22)	667 (57.48)	Ref	Ref	–	–
Currently Working	86 (45.6)	146 (47.78)	438 (42.52)	1.09 (0.51 to 2.36)	0.88 (0.51 to 1.51)		
Place of residence							
Rural	158 (52.86)	273 (41.69)	785 (31.43)	Ref	Ref	Ref	Ref
Urban	146 (47.14)	389 (58.31)	1440 (68.57)	1.57 (0.92 to 2.68)	2.45 (1.48 to 4.05)*	1.53 (0.91 to 2.58)	2.16 (1.37 to 3.41)*
MPCE quintile							
Poorest	44 (17.84)	89 (13.26)	255 (9.198)	Ref	Ref	–	–
Poorer	51 (16.87)	82 (11.68)	321 (12.96)	0.93 (0.38 to 2.31)	1.49 (0.66 to 3.36)		
Middle	53 (13.57)	123 (25.89)	405 (12.98)	2.57 (0.88 to 7.50)	1.85 (0.81 to 4.25)		
Richer	69 (22.09)	142 (21.85)	548 (29.03)	1.33 (0.54 to 3.28)	2.55 (1.00 to 6.47)*		
Richest	87 (29.63)	226 (27.31)	696 (35.83)	1.24 (0.52 to 2.93)	2.34 (0.98 to 5.62)		
BMI (kg/m^2^) (n=3190)							
Underweight/Normal	21 (6.56)	64 (10.47)	178 (5.25)	Ref	Ref	–	–
Overweight	41 (16.75)	107 (17.82)	297 (10.32)	0.67 (0.24 to 1.89)	0.77 (0.29 to 2.03)		
Obese	241 (76.69)	491 (71.71)	1750 (84.43)	0.59 (0.27 to 1.26)	1.38 (0.69 to 2.76)		
Smoking status (n=241)							
No	14 (34.19)	29 (48.04)	89 (57.57)	Ref	Ref	–	–
Yes	19 (65.81)	26 (51.96)	64 (42.43)	0.56 (0.16 to 1.92)	0.38 (0.14 to 1.04)		
Alcohol consumption (n=3186)							
No	257 (88.83)	593 (92.41)	2021 (93.23)	Ref	Ref	–	–
Yes	47 (11.17)	68 (7.594)	200 (6.771)	0.65 (0.33 to 1.30)	0.58 (0.31 to 1.07)		
Physical activity (n=3185)							
Frequently	60 (17.65)	124 (27.6)	367 (17.61)	Ref	Ref	–	–
Rarely	17 (5.685)	54 (8.241)	122 (4.465)	0.93 (0.32 to 2.66)	0.79 (0.32 to 1.91)		
Never	226 (76.67)	483 (64.16)	1732 (77.92)	0.53 (0.24 to 1.19)	1.02 (0.53 to 1.95)		
Media exposure (n=3176)							
No	58 (24.58)	89 (20.07)	272 (12.56)	Ref	Ref	Ref	Ref
Yes	246 (75.42)	568 (79.93)	1943 (87.44)	1.30 (0.74 to 2.28)	2.27 (1.32 to 3.91)*	1.20 (0.72 to 2.01)	1.92 (1.19 to 3.11)*
Additional comorbidities							
None	198 (65.38)	361 (53.97)	1226 (46.5)	Ref	Ref	Ref	Ref
1 or more	106 (34.62)	301 (46.03)	999 (53.5)	1.61 (0.95 to 2.73)	2.17 (1.28 to 3.70)*	1.41 (0.83 to 2.42)	1.95 (1.22 to 3.10)*

Crude RRR1=partial Ttreatment versus Nno Ttreatment, Ccrude RRR2=full Ttreatment versus Nno Ttreatment.

*P<0.05

BMI, body mass index; MPCE, monthly per capita expenditure; RRR, relative risk ratio

Additionally, we performed a binary logistic regression by combining the categories of no and partial treatments and comparing it with full treatment (online supplemental table 3). On adjusted analysis, having secondary education, urban residence, richest MPCE quintile and obesity were the significant predictors associated with being on full treatment as compared with being on no or partial treatment.

Among the participants having metabolic syndrome, the following factors were significantly associated with no treatment coverage for both DM and HTN comorbidities: age group 45–59 years compared with older participants (χ^2^=17.76, p<0.001), those working compared with non-working participants (χ^2^=6.38, p=0.012), rural residence compared with urban residence (χ^2^=28.13, p<0.001), having no media exposure compared with media exposure (χ^2^=10.08, p=0.001) and those having no additional comorbidity compared with those with additional comorbidities (χ^2^=9.02, p=0.003).

## Discussion

This study evaluated the country and regional prevalence of metabolic syndrome in individuals in India aged over 45 years and ascertained their biomedical and psychosocial risk factors with their diagnoses and treatment statuses. The gross prevalence of metabolic syndrome in this study (4.83%) is much lower compared with certain studies conducted in the northern (28.6%),^30^ and southern (45.9%)^31^ regions of India, and also the pooled estimates from a meta-analysis of both facility and community based studies conducted in India (30.0%).^25^ The lower prevalence of metabolic syndrome observed in this study is likely an underestimate due primarily to over-reliance on self-reporting in the context of poor rates of diagnosis of DM and hypercholesterolaemia.^32^ Alternative factors may also have contributed, such as lack of representativeness of smaller studies, variable application and definition of the diagnostic criteria of metabolic syndrome and temporal changes in prevalence. However, the data presented here and in previous studies suggest that only a small proportion of individuals with metabolic syndrome in India are diagnosed.^25^

Despite underestimating prevalence, this study found a concerningly low rate of full treatment for the component conditions of metabolic syndrome. The risk factors associated with lack of provision of treatment identified here were middle-age (45–59 years), working status (specifically, being in employment), rural residence, no media exposure and no additional comorbidities. These patients should be specifically considered when designing public health interventions to improve cardiovascular health by addressing the burden of metabolic syndrome.

This study observed the prevalence of metabolic syndrome in females as much higher than males, a finding consistent with prior studies^26 33^ despite a comparatively higher epidemiological burden of undetected cases of DM and HTN in this subgroup.^34 35^ Female sex with increasing age may be a risk of metabolic syndrome due to oestrogen deficiency during menopause which is hypothesised to contribute to insulin resistance, abdominal obesity, high triglycerides and reduced HDL-c levels.^36^ In this study, the odds of having metabolic syndrome increased up to the age of 79 years and then underwent a sharp decline, a finding in line with that from the Singaporean Healthy Older People Everyday study.^37^ This interruption in the trend may represent a selection bias as individuals with metabolic syndrome are more likely to die earlier as a consequence of their poorer health. Fewer than 1% of the participants exhibited all four component conditions of metabolic syndrome.

As per this study, the burden of metabolic syndrome is higher in urban areas relative to rural areas in India; a finding consistent with evidence from studies in China^38^ and Bangladesh.^39^ This may be due to urban populations exhibiting less physical activity and unhealthier dietary habits involving increased polished rice and processed food consumption.^40 41^ This study also observed a statistically significant association between increasing BMI and the occurrence of metabolic syndrome, a finding corroborating evidence from previous studies in India.^42 43^ Within the high-risk subgroup, this study observed a lack of association between alcohol consumption and metabolic syndrome, despite prior evidence showing significant positive correlation^44^ As smoking was negatively associated with metabolic syndrome prevalence—contradicting strong biological and epidemiological evidence—it is possible that lifestyle variables were compromised by patient embarrassment or denial.^45^

This study also found that nearly one in four individuals with metabolic syndrome who had previously been diagnosed with DM and HTN were currently not taking treatment, suggesting that poor medication adherence (rather than complete lack of provision of healthcare) may be a significant factor.^46 47^ Low medication adherence in patients with DM and HTN with poor resultant glycaemic and blood pressure control have been reported extensively in India.^20 48^ However, alternative reasons for low adherence may relate to healthcare infrastructure failing to provide accessible and affordable treatment for patients, and this study did not explore the specific reasons for observed low treatment rates. Consequently, the design and promotion of effective interventions to improve medication provision and adherence among patients with metabolic syndrome are warranted to prevent and reduce their risk and burden of cardiovascular complications.^49^ This study suggests that patients of metabolic syndrome with difficult health accessibility such as those living in rural areas, and those lacking exposure to mass media with possibility of reduced awareness are at significantly higher risk of lacking treatment necessitating the need for improving treatment awareness, accessibility and affordability for patients in resource-limited settings. Community-health volunteers and professionals have previously been shown to improve medication adherence in India, but research and implementation in the specific context of metabolic syndrome is limited.^50^ In this context, ongoing schemes aiming to provide pharmacological treatment to 75 million HTN patients by 2025 may be unrealistic; and plans to instil lifestyle modifications must contend with limited access to healthier food and opportunities for recreational exercise associated with lower socioeconomic settings.^51^ ^52 53^

The major strengths of this study were its large sample size and national-level representation of older Indian adults. Despite these strengths, the study exhibited four main limitations. First, the cross-sectional design precluded inference of causal relationships. Further investigation is required to establish the root causes of metabolic syndrome prevalence and treatment provision varying significantly across various risk factors. Second, this study only investigated older adults aged over 45 years, and a significantly proportion of metabolic syndrome patients were, therefore, likely excluded.^54^ Third, because no data were available for blood glucose and cholesterol levels, DM and hypercholesterolaemia status was based solely on self-reporting, which likely led to underreporting of the prevalence of metabolic syndrome due to limited access to investigations as well as recall bias.^55^ Moreover, the degree of blood pressure control, lipid control and insulin resistance could not be ascertained due to reliance on self-reported data without access to longitudinal results of investigations, limiting conclusions concerning treatment status. Fourth, to comprehensively establish the burden of metabolic syndrome, further research is required to determine the economic cost of disease in India with consideration of direct, indirect and intangible costs.

Although our study reported a comparatively lower prevalence—with low confidence—of metabolic syndrome in India, it also highlighted higher prevalence of the subcomponents of metabolic syndrome, indicating that focused prevention, early detection of risk factors and treatment of each component is necessary to reduce associated mortality and morbidity in older adults. Our findings also illuminate potentially fruitful avenues for policy development. Major initiatives are necessary to identify individuals with metabolic syndrome—perhaps focusing on those at highest risk—and subsequently intervene with well-evidenced medications to reduce rates of cardiovascular mortality and morbidity. Interventions may include education initiatives, large-scale blood test screening and improved provision of healthcare advice and access to medical treatment. Systematic, cost-effective programmes with effective planning and implementation are required. Interdisciplinary collaborations across health and local government, as well as effective utilisation of available resources and wider community involvement may all contribute to better outcomes for the population.

## supplementary material

10.1136/bmjph-2023-000389 online supplemental file 1

## Data Availability

Data are available in a public, open access repository. cited above.
