# Supplementary material for: Burden, determinants and treatment status of metabolic syndrome among older adults in India: a nationally representative, community-based cross-sectional survey
Source: BMJ Public Health. 2023 Nov 20;1(1):e000389. doi: 10.1136/bmjph-2023-000389 (PMC11812726; doi:10.1136/bmjph-2023-000389)
Supplement: online supplemental file 1 [file bmjph-1-1-s001.pdf]

Supplementary Table 1: Potential predictive variables for which data were collected

Category	Variable	Classifications
**Biomedical**	Sex	Male; female
	Age in years	45-59; 60-69; 70-79; 80+
	BMI	Underweight (<18.5); normal (18.5-22.9); overweight (23-24.9); obese (25.0+)
	Physical activity	Frequent (every day or more than once per week); rare (once per week or 1-3 times per month); never (hardly ever or never).
	Smoking status	Smoker; non-smoker
	Alcohol consumption	Drinker; non-drinker
	Additional comorbidities	Self-reported presence of medical conditions
**Psychosocial**	Education	None/less than primary; primary completed; secondary completed; higher/graduate
	Working status	Currently working; not currently working
	Marital status	Never married; currently married; widowed/divorced/separated/deserted
	MPCE	Richest to poorest quintiles
	Media exposure	Yes; no
	Place of residence	Urban; rural

Abbreviations: BMI, body-mass index; MPCE, monthly per capita expenditure

Supplementary Table 2: State-wise performance indicators and prevalence of metabolic syndrome

State	Total	Individuals with MetS	Weighted Prevalence % [95% CI]
Andaman and Nicobar Islands	1,113	79	7.63[5.88,9.85]
Andhra Pradesh	2,399	167	7.02[6.00,8.21]
Arunachal Pradesh	982	11	1.48[.61,3.54]
Assam	2,019	20	1.15[.68,1.93]
Bihar	3,297	73	2.50[1.80,3.47]
Chandigarh	932	117	11.53[9.41,14.05]
Chhattisgarh	100	40	2.27[1.62,3.18]
Dadra and Nagar Haveli	989	27	4.54[3.03,6.76]
Daman and Diu	903	75	9.31[7.113,12.09]
Delhi	1,171	100	8.18[6.51,10.23]
Goa	1,265	142	11.05[9.15,13.28]
Gujarat	2,145	106	5.82[4.70,7.19]
Haryana	1,757	64	3.75[2.83,4.94]
Himachal Pradesh	1,255	87	7.48[5.79,9.60]
Jammu and Kashmir	1,486	88	6.34[4.76,8.40]
Jharkhand	2,228	42	1.96[1.40,2.74]
Karnataka	2,083	120	7.79[4.54,13.06]
Kerala	2,300	453	19.76[17.80,21.88]
Lakshadweep	1,062	168	15.86[13.23,18.91]
Madhya Pradesh	2,717	69	2.75[2.00,3.77]
Maharashtra	3,592	209	4.77[3.97,5.72]
Manipur	1,251	51	4.32[3.10,5.98]
Meghalaya	886	15	1.86[1.07,3.22]
Mizoram	1,129	30	3.23[2.19,4.72]
Nagaland	1,202	15	2.99[1.21,7.22]

Odisha	2,621	75	2.91[2.29,3.68]
Puducherry	1,288	168	11.50[9.55,13.79]
Punjab	1,972	236	12.44[10.75,14.35]
Rajasthan	2,131	50	2.28[1.66,3.12]
Sikkim	1,044	31	3.87[2.63,5.68]
Tamil Nadu	3,205	248	6.33[5.41,7.40]
Telangana	2,252	111	5.60[4.60,6.80]
Tripura	1,044	29	2.77[1.87,4.08]
Uttar Pradesh	1,262	96	2.27[1.80,2.86]
Uttarakhand	4,289	54	4.13[3.12,5.46]
West Bengal	3,434	164	4.13[3.28,5.20]
**Overall Prevalence**	**66,606**	**3,630**	**4.91[4.61,5.22]**

Supplementary Table 3: Determinants of treatment-seeking behaviour among participants with metabolic syndrome using binary logistic regression[1] (N = 3191)

Variables	Not on Treatment n (weighted %) (N = 304)	Partial Treatment n (weighted %) (N = 662)	Full Treatment n (weighted %) (N = 2225)	Crude OR [95% CI]	Adjusted OR[2] [95% CI]
**Age (years)**					
45-59	166 (54.56)	300 (35.56)	886 (37.25)	Ref	-
60-69	90 (29.81)	233 (42.68)	869 (40.56)	1.19 [0.71, 2.00]	
≥70	48 (15.64)	129 (21.76)	470 (22.20)	1.27 [0.65, 2.49]	
**Sex**					
Male	73 (20.82)	174 (30.75)	566 (22.23)	Ref	-
Female	231 (79.18)	488 (69.25)	1659 (77.77)	1.32 [0.78, 2.25]	
**Education (n = 2222)**					
No education or less than primary	33 (17.76)	97 (25.86)	264 (13.73)	Ref	Ref
Primary complete	58 (28.73)	104 (17.75)	379 (19.29)	1.55 [0.93, 2.56]	1.39 [0.86, 2.26]
Secondary	70 (32.49)	176 (29.14)	596 (49.25)	2.77 [1.43, 5.38] *	1.90 [1.12, 3.21] *
Higher	17 (6.10)	45 (8.423)	131 (6.006)	1.33 [0.73, 2.42]	1.05 [0.56, 1.97]
Graduate and above	19 (14.92)	48 (18.84)	185 (11.73)	1.13 [0.39, 3.28]	0.76 [0.30, 1.91]
**Marital status**					
Never married	1 (0.0567)	8 (0.4616)	14 (0.3711)	Ref	-
Currently married	215 (69.65)	476 (72.93)	1537 (62.8)	0.77 [0.18, 3.24]	
Widowed/ Divorced/ Separated/ Deserted	88 (30.29)	178 (26.6)	674 (36.83)	1.16 [0.26, 5.29]	
**Work Status (n = 1645)**					

Not working	83 (54.4)	225 (52.22)	667 (57.48)	Ref	-
Currently Working	86 (45.6)	146 (47.78)	438 (42.52)	0.83 [0.49, 1.42]	
**Place of residence**					
Rural	158 (52.86)	273 (41.69)	785 (31.43)	Ref	Ref
Urban	146 (47.14)	389 (58.31)	1440 (68.57)	1.82 [1.20, 2.74] *	1.78 [1.20, 2.64] *
**MPCE Quintile**					
Poorest	44 (17.84)	89 (13.26)	255 (9.198)	Ref	Ref
Poorer	51 (16.87)	82 (11.68)	321 (12.96)	1.55 [0.94, 2.56]	1.58 [0.86, 2.89]
Middle	53 (13.57)	123 (25.89)	405 (12.98)	0.96 [0.50, 1.86]	0.79 [0.37, 1.68]
Richer	69 (22.09)	142 (21.85)	548 (29.03)	2.13 [1.12, 4.05] *	1.68 [0.86, 3.28]
Richest	87 (29.63)	226 (27.31)	696 (35.83)	2.05 [1.14, 3.69] *	1.91 [1.02, 3.56] *
**BMI (kg/m^2) (n = 3190)**					
Underweight/Normal	21 (6.56)	64 (10.47)	178 (5.25)	Ref	Ref
Overweight	41 (16.75)	107 (17.82)	297 (10.32)	1.03 [0.59, 1.82]	0.98 [0.46, 2.09]
Obese	241 (76.69)	491 (71.71)	1750 (84.43)	2.01 [1.21, 3.35] *	1.94 [1.01, 3.73] *
**Smoking status (n = 241)**					
No	14 (34.19)	29 (48.04)	89 (57.57)	Ref	-
Yes	19 (65.81)	26 (51.96)	64 (42.43)	0.56 [0.25, 1.25]	
**Alcohol consumption (n = 3186)**					
No	257 (88.83)	593 (92.41)	2021 (93.23)	Ref	-
Yes	47 (11.17)	68 (7.594)	200 (6.771)	0.75 [0.48, 1.18]	
**Physical activity (n = 3185)**					
Frequently	60 (17.65)	124 (27.6)	367 (17.61)	Ref	-
Rarely	17 (5.685)	54 (8.241)	122 (4.465)	0.83 [0.41, 1.69]	
Never	226 (76.67)	483 (64.16)	1732 (77.92)	1.57 [0.83, 2.96]	

**Media exposure (n = 3176)**					
No	58 (24.58)	89 (20.07)	272 (12.56)	Ref	Ref
Yes	246 (75.42)	568 (79.93)	1943 (87.44)	1.92 [1.22, 3.02] *	1.32 [0.78, 2.25]
**Additional comorbidities**					
None	198 (65.38)	361 (53.97)	1226 (46.5)	Ref	-
1 or more	106 (34.62)	301 (46.03)	999 (53.5)	1.58 [0.99, 2.51]	

Abbreviations: MPCE, monthly per capita expenditure; BMI, body mass index; OR, odds ratio; CI, confidence interval

[1]Binary logistic regression performed by combining no and partial treatment versus full treatment

[2]Variables found to be significant (P<0.05) in crude analysis were added to the final adjusted model

*P<0.05, **P<0.001

Goodness of fit: P = 0.6248
